# Cultural Bias in Parent Reports: The Role of Socialization Goals When Parents Report on Their Child’s Problem Behavior

**DOI:** 10.1007/s10578-022-01464-y

**Published:** 2022-11-12

**Authors:** Ronja A. Runge, Renate Soellner

**Affiliations:** Department of Psychology, University of Hildesheim, Hildesheim, Germany

**Keywords:** Cultural bias, Comparability, Parent report, Child mental health, Socialization goals

## Abstract

It has been called into question whether widely used screening instruments for child mental health can provide comparable results across countries and cultures. Socialization goals can influence whether and to what extent a parent considers a behavior to be problematic and thus might influence parental reports on their child’s behavior. We tested comparability of parental reports between native German (*N* = 116) and Turkish origin (*N* = 77) parents in Germany in an online study using a vignette approach. Parents were asked to rate the perceived problem severity of the same behavior depicted in the vignettes. We expected and found that parents of Turkish origin in Germany rate the externalizing problem behaviour depicted in the vignettes as more problematic compared to native German parents. The effect was fully mediated by parental approval of the socialization goals obedience and collectivism. We also controlled for social desirability responding and an extreme response style.

**Supplementary Information:**

The online version contains supplementary material available at 10.1007/s10578-022-01464-y.

Worldwide, the number of international immigrants has increased continuously over the past five decades (from 84 million in 1970 to 281 million in 2020). In 2021, Germany was the second top destination country for immigrants [1] and about 40% of children under the age of 10 years living in Germany were 1st (7.2%) or 2nd or 3rd generation (32.8%) immigrants [2]. Being an immigrant might be a risk factor for children’s mental condition, thus monitoring the mental health of immigrant children is a societal task [3].

For younger children in general, parent reports are used to assess their mental health status. In Germany, immigrant parents reported more emotional and behavioral problems among their children compared to native German parents in various studies [4–6]. These studies used widely known screening instruments for children’s mental health like the strengths and difficulties questionnaire (SDQ, [7]) and the child behavior checklist (CBCL, [8]).

However, some authors have questioned whether these instruments provide cross-culturally comparable scores [9–11]. Stevanovic et al. [11] reviewed studies examining cross-cultural measurement invariance and differential item functioning of a variety of children and adolescent psychopathology scales. They concluded that most assessment instruments show weak or no measurement invariance. In Germany, Runge and Soellner [10] found missing measurement invariance when comparing SDQ parent reports between native German, Turkish Origin and Russian origin parents. Other studies also challenged the comparability of scores: Heiervang and colleagues [9] used the SDQ as a screening instrument and an additional clinical diagnostic interview to check the validity of SDQ scores. They found that Norwegian parents underreported emotional problems compared to British parents when using the SDQ. Bevaart et al. [12] found that, amongst children with equally high parental ratings of SDQ scores, ethnic-minority parents perceived their child’s behavior as less problematic than ethnic-majority parents in the Netherlands. Thus, ethnic-minority parents might state higher SDQ scores than ethnic-majority parents when their child’s problem behavior is equally severe.

Using vignettes is another promising approach when testing for comparability of parental reports. Vignettes are “brief texts depicting hypothetical individuals who manifest the trait of interest (e.g., health) to a lesser or greater degree” [13]. Parents thus rate the problem severity of the described behavior in the vignettes irrespective of their own child’s behavior. Differences in problem severity ratings indicate response bias. Using this approach, differences in the ratings of child problem behavior vignettes between American and Thai parents [14], American and Jamaican parents [15], African and European American adults [16] and Latino and Anglo American parents [17] have been found. So far, reasons for this difference in recognition, classification and reporting of children’s problem behavior among different cultures or ethnic groups have not been explored systematically.

## Socialization Goals

What parents consider to be a problematic behavior might be influenced by their socialization goals [15, 16]. Socialization goals are one aspect of parenting beliefs. They refer to the characteristics parents value and want their children to attain when they grow up [18, 19]. Socialization goals are informed by the cultural model of the parents [20]. According to Kagitcibasi and Ataca [21], two dimensions form the cultural model: interpersonal distance (with the poles of relatedness and separateness) and agency (with the poles of autonomy and heteronomy). Western societies are often characterized as separated and autonomous, which is called the independent model [22]. Parents in these societies value self-enhancement and self-maximization in their child [20]. The second cultural model is the interdependent model. In these collectivistic societies (often in rural, subsistence-based families), individuals are perceived as interrelated and heteronomous. Parents in these societies are more likely to value obedience in their child [20]. The third model, the model of autonomous relatedness, can be found in urban, educated, middle-class families from societies with an interrelated cultural heritage which have undergone socio-economic development [22]. Kagitcibasi and Ataca [21] investigated adolescents, their mothers and their grandmothers in Turkey and showed that while material interdependencies decreased with socioeconomic development in the last decades, psychological interdependencies did not. Families in autonomous relatedness societies value obedience less and autonomy more than families in interdependent societies, but close family ties continue to be important.

How much parents value socialization goals like obedience, self-enhancement and harmonious relationships influences how they evaluate their child’s behavior. In the US, due to the high value of collectivism in the African American community, African American parents might rate externalizing behavior as more serious than internalizing behavior [16]. Externalizing problem behavior has a direct impact on the child's environment. It is disruptive to the family or the neighborhood. It thus contradicts collectivist values such as "conforming" or "standing back" more than internalizing problem behavior does. Externalizing behavior accordingly violates the cultural norm of this group more severely than internalizing behavior. The same can be expected for the value of obedience. Externalizing problem behaviors, like aggressive behaviors or stealing, are likely to contradict how parents want their children to behave. Thus, the more important it is for the parent that the child does as he/she is told, the more severe such a behavior is likely to be rated. While we expect collectivism and obedience to influence how parents evaluate externalizing problem behavior, we do not expect them to influence how parents evaluate internalizing problem behavior. Self-development is another important socialization goal, more pronounced in societies characterized as independent, but also valued in societies assigned to the autonomous relatedness model [20]. Self-development as a socialization goal could be positively correlated to how parents perceive internalizing problem behavior. The rationale is that a child’s anxiety and depressive symptoms (internalizing problems) stand in the way of the child expressing him- or herself, as well as being curious and self-confident (self-development goals).

Mainstream German society can be characterized as independent. Accordingly, parents highly value self-enhancement and self-maximization. Immigrant parents in Germany however might additionally value socialization goals, which are important in their countries of origin. Turkish immigrants in Germany, who form the largest group of immigrants in Germany [2], came to Germany in the 1960s as part of a guest worker agreement between Germany and Turkey. At that time, the majority of Turkish immigrants had a low level of education and came from rural areas, that is, from an interdependent society. However, Turkish society has changed strongly since then. In the last 5 years, there was a high share of highly educated immigrants from Turkey to Germany [23]. Being educated and coming from an urbanized country, recent Turkish immigrants can be assigned to the autonomous-related model. They are thus likely to value obedience less and autonomy more than families in interdependent societies, while continuously valuing close family ties.

There are a few studies investigating the socialization goals of Turkish immigrant parents in Germany. Turkish origin parents were found to value obedience and close family ties more and autonomy less than native German parents in Germany [18, 24–27]. How long the parents had stayed in Germany and if they were born in Turkey or in Germany (2nd generation immigrants) did little to predict socialization goals [26]. This might be explained by immigrants having close contacts within the Turkish community, living in neighborhoods with many other Turkish immigrants or engaging in Turkish organizations in their free time in Germany [26]. Adaption to Germany in Turkish mothers was otherwise correlated with less valuation of obedience and more autonomy, while still highly valuing close family relations [18].

## Social Desirability Responding and Extreme Responding

Further sources of bias in reporting behavior which could cause missing comparability in parental reports between different cultural groups are social desirability responding and response styles. Social desirability responding has been shown to be correlated to parental reports of their child’s problem behavior [28] and varies between cultures [29]. There is evidence that social desirability is stronger in collectivist cultures [29].

Response styles, like extreme responding (the tendency to choose the most extreme options in a scale) differ between cultures and countries [30–32]. Lamm and Keller [33] examined the relation between extreme responding and cultural models. They found a “tendency that respondents from cultural environments regarded as more collectivistic/interdependent show more extreme responding than respondents from cultural environments regarded as more individualistic/independent. Mothers with an autonomous-related sociocultural orientation responded still more extremely than mothers with an independent sociocultural orientation (…)” [33].

### Hypotheses

The aim of the present study is to investigate whether Turkish immigrant parents rate externalizing problem behavior as more problematic than native German parents. Furthermore, we wanted to test whether this is due to a higher appreciation of the socialization goals obedience and collectivism. Since previous research has shown that Turkish preschool children are often treated with indulgence and misbehavior is treated with great tolerance [24], we expect a difference in ratings only for school children.

The following hypotheses, the study design and the instruments were preregistered in the Open Science Framework (OSF) previous to data collection (https://doi.org/10.17605/OSF.IO/ZBQU3; we originally planned to analyze Russian origin parents as well, but due to a very small sample size, we had to refrain from that plan):

1. Turkish origin and native German parents differ in their rating of the vignettes. Turkish origin parents rate the behavior described in the vignettes as more problematic than native German parents when they think of their school child.
  - This difference is more pronounced when rating the problem severity of externalizing problems compared to internalizing problems.
  - There is no difference in the problem severity rating when they think of their preschool child.
2. The relation between cultural origin and the problem severity rating of externalizing problem vignettes is partly mediated by the socialization goals obedience and collectivism.
  - Parents of Turkish origin agree more strongly with the socialization goals obedience and collectivism.
  - Parents, who value obedience and collectivism more in their children, rate the behavior in the externalizing problem vignettes as more problematic.

Since previous research has highlighted the role of social desirability and extreme responding for response behavior, we include both constructs in the mediation analysis. We thus ensure that the expected mediation by socialization goals is not a spurious mediation and the differences in the problem severity ratings between the groups are not only found because of differences in social desirability or extreme responding.

We additionally exploratorily test if immigrant generation (1st vs. 2nd generation) or length of stay in Germany is correlated to problem severity ratings, and how the socialization goal self-development influences severity ratings of internalizing problem behavior.

## Methods

### Procedure

The ethics committee of the University of Hildesheim approved the study. Target groups were native German and Turkish origin parents of children between 4 and 12 years of age in Germany. We decided for that age range for two reasons: (1) Since youth self-reports are not available up to 11 years of age, parental reports are particularly important, (2) the same vignettes should be used for all participants. As the kind of problem behavior shown in infancy and later adolescence is differing hugely, no comprehensive behavior descriptions could have been provided. Participants were recruited online (social media, forums, contacting schools and kindergartens as well as cultural associations per mail) and via notices (in restaurants, supermarkets, and at pediatricians). All participants agreed to take part in the survey and confirmed that they had read the privacy statement at the beginning of the survey. The online survey took about 15 min to complete. As an incentive, vouchers (50 €) for an online shop were raffled.

### Translation of Study Material

Socialization goal scales were translated from English to German and back translated to English by a native speaker. All other study material already existed or were developed in German. All materials were translated to Turkish by a native speaker. Back translation was performed by another native speaker. In the case of discrepancies, the process was repeated.

### Measures

#### Cultural Origin

Respondents were allocated to the native German group if they and both of their parents were born in Germany, if they had German citizenship and if they spoke German at home. Respondents were allocated to the Turkish origin group if they and both parents had been born in Turkey, if they had Turkish citizenship or if they spoke mainly Turkish at home.

#### Socio-Demographic Information

Respondents were asked the number, age and gender of children, their own gender and age, years of education, marital status, residential area (urban or rural), country of birth, country of birth of their parents, citizenship, language at home, and if applicable: length of stay in Germany.

#### Immigrant Generation

Respondents born abroad were classified as 1st generation immigrants. Respondents born in Germany whose parents were born abroad were classified as 2nd generation immigrants.

#### Socialization Goals

Obedience was measured using four items from the obedience socialization goal scale in the German Socio-economic Panel [34]. Collectivism was measured by the Collectivism socialization goal scale by Li et al. [35] with five items. Additionally, the socialization goal self-development (four items) developed by Chao [36] was used. Response format was a 5-point Likert scale (*not important at all* to *very important*).

#### Vignettes

Six vignettes depicting problem behavior of children were developed for this study. The described behavior in the vignettes was oriented towards the problem behavior which is queried in the Child Behavior Checklist [8]. Three vignettes depicted internalizing behavior (mild, moderate, severe degree) and three vignettes depicted externalizing behavior (mild, moderate, severe degree). For example, for severe externalizing problem behavior (English translation): “In the last 2 months, your child has mostly not done what you told him/her to do. He/she has repeatedly broken things of other children and of yours on purpose and has hit other children in different situations. Punishment and talking did not change his/her behavior”. Parents were asked to imagine their own child showing this behavior and then to rate on a seven-point Likert scale how problematic they would find this behavior. If respondents had more than one child between 4 and 12 years of age, they were asked to imagine the child whose birthday is earlier in the year (regardless of age).

Vignettes were pretested by experts (three children and youth therapists, a primary school teacher, a kindergarten teacher) and 6 mothers (three native German and three Turkish mothers). The vignettes were rated as easy to understand and the expected ranking of the severity of the problem behavior was confirmed.

#### Social Desirability

The KSE-G [37] was used to measure Social Desirability with six items on a 5-point Likert scale.

#### Extreme Response Style

Extreme response style was measured by counting the number of times the lowest or highest answer choice was chosen by the respondent in all scales of the study.

### Statistical Analysis

Only respondents who stated that they had a child between 4 and 12 years of age and only those allocated to the native German or the Turkish origin group were included in the analyses.

For all of the following analyses, except the parallelization, the *lavaan* package in *R* [38] was used. Robust maximum likelihood estimation was used (RML) for all of the following CFA and SEM analyses, Full information maximum likelihood estimation (FIML) was used for missing data. The parallelization was done using the IBM statistical package of social sciences (SPSS) version 26.0 for Windows.

#### Preliminary Analyses

In order to minimize the influence of other potentially confounding variables, the native German and the Turkish Origin sample were parallelized by gender and age of the child, university degree, gender and age of the respondent, partnership status and urban/rural residential area. In each stratum (e.g., male child) in the larger native German sample, a random sample was drawn with equal sample-size as in the corresponding stratum in the Turkish Origin group. After matching, there were no significant differences in the aforementioned characteristics between the group of native German parents (*N* = 116) and Turkish Origin parents (*N* = 77).

To ensure valid comparisons between groups, measurement invariance was tested using multi group confirmatory factor analysis (MG CFA). We used χ^2^, the comparative fit index (CFI) and the root mean square error of approximation (RMSEA) to evaluate the model fit. A CFI > 0.90 was rated as acceptable and > 0.95 as good, a RMSEA < 0.6 was rated as good [39]. To evaluate the meaningfulness of changes of the model fit we used the change in the CFI (ΔCFI) because this index is proposed to be independent of overall model fit and sample size. A value of ΔCFI smaller than or equal to –(0.01 indicates that the null hypothesis of invariance should not be rejected [40]. In the case of partial invariance, we modelled regression paths from group to the non-invariant items in the mediation analyses (analogous to the multiple indicator multiple causes (MIMIC) approach).

#### Main Analyses

Regarding ratings of problem severity, a mean of the three internalizing problem behavior vignettes and the three externalizing problem behavior ratings was calculated for the following analyses.

To test the first hypothesis, a path model was analyzed with group (native German parent or Turkish origin parent) as predictor, mean rating of internalizing and externalizing problem behavior vignettes as outcome and age of the child (preschool child/school child) as moderator.

To test the second hypothesis, two mediation models were analyzed with group (native German parent or Turkish origin parent) as predictor, socialization goals obedience or collectivism and social desirability as latent mediators and extreme responding as a manifest mediator. Since the KSE-G (social desirability) has positively and negatively worded items, we included a method factor for the positively worded items. Mean rating of externalizing problem behavior vignettes served as outcome.

#### Additional Analyses

For the socialization goal self-development, a mediation model was built with group as the predictor, self-development, social desirability and extreme responding as mediators and the problem severity rating of the internalizing vignettes as the outcome. To test for the impact of length of stay and immigrant generation at problem severity ratings, a Pearson correlation for length of stay and a product moment correlation for immigrant generation was calculated.

## Results

### Descriptive Statistics

Sociodemographic characteristics for the parallelized samples can be found in Table 1. Bivariate correlations of latent (obedience, collectivism, self-development, social desirability) and manifest variables can be found in Table A1 in the online supplementary material. In the Turkish origin parent sample, most respondents were 1st generation immigrants (83.1%) with a mean length of stay in Germany of 7.11 years (SD = 7.66 years).

**Table 1:** Sociodemographic characteristics after parallelization

	Group		*χ*^2^-Test
	Native German parents (*N* = 116)	Turkish origin parents (*N* = 77)	
	*N*	*N*	
Gender of the child (female)	64 (55.7%)	33 (44.6%)	*χ*^*2*^(1) = 2.2, *p* = 0.14
Gender of the respondent (female)	110 (96.5%)	74 (98.7%)	*χ*^*2*^(1) = 0.831, *p* = 0.36
University degree	90 (77.6%)	59 (86.8%)	*χ*^2^(1) = 2.35, *p* = 0.13
Living in an urban residential area (vs. rural)	93 (80.2%)	66 (90.4%)	*χ*^*2*^(1) = 3.52, *p* = 0.06
Age of the child: school child (vs. preschool child)	70 (60.9%)	51 (68.0%)	*χ*^2^(1) = 1.0, *p* = 0.32

### Measurement Invariance of Socialization Goal Scales

Obedience showed strong measurement invariance between groups. Collectivism and self-development showed partial invariance. Results are shown in the online supplementary material (Table A2 to A5).

### H1: Difference in Ratings of Vignettes Between Native German and Turkish Origin Parents

Turkish origin parents rated the vignettes describing externalizing behavior as more severe than native German parents (*B* = 1.85, *p* = 0.03). There was no difference in the ratings of vignettes describing internalizing behavior (*B* = − 0.22, *p* = 0.78). There was no main effect of age of the child on the rating of internalizing behavior vignettes (*B* = 0.06, *p* = 0.89) or externalizing behavior vignettes (*B* = − 0.08, *p* = 0.91). There was also no interaction effect of age of the child and group on the vignette ratings (*B* = − 0.53, *p* = 0.28 and *B* = 0.06, *p* = 0.89).

### H2: Mediation

**Fig. 1:**
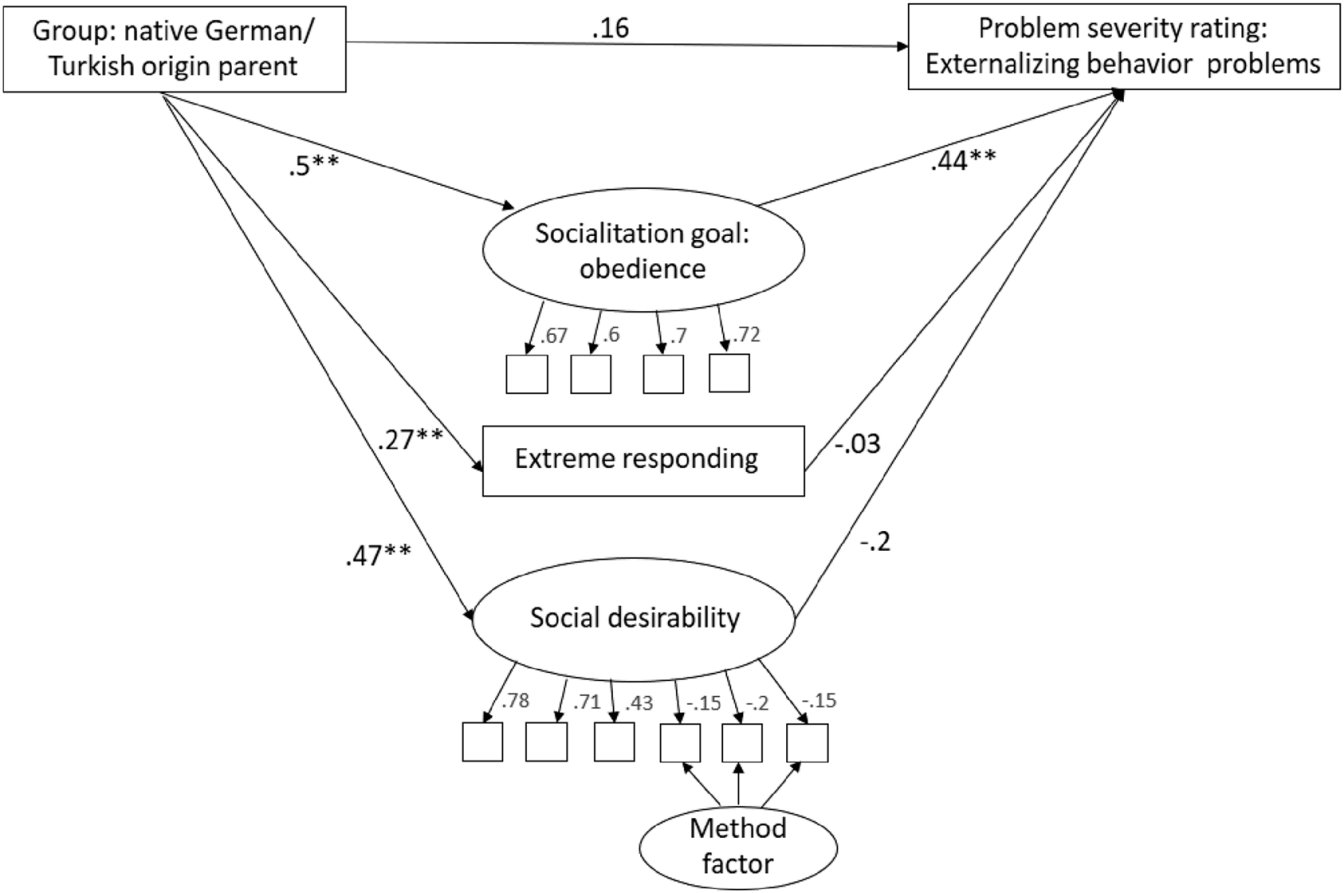
Mediation model with obedience as mediator. Standardized path coefficients (*β*) are dispayed. **p  < 0.01; *p  < 0.05

**Fig. 2:**
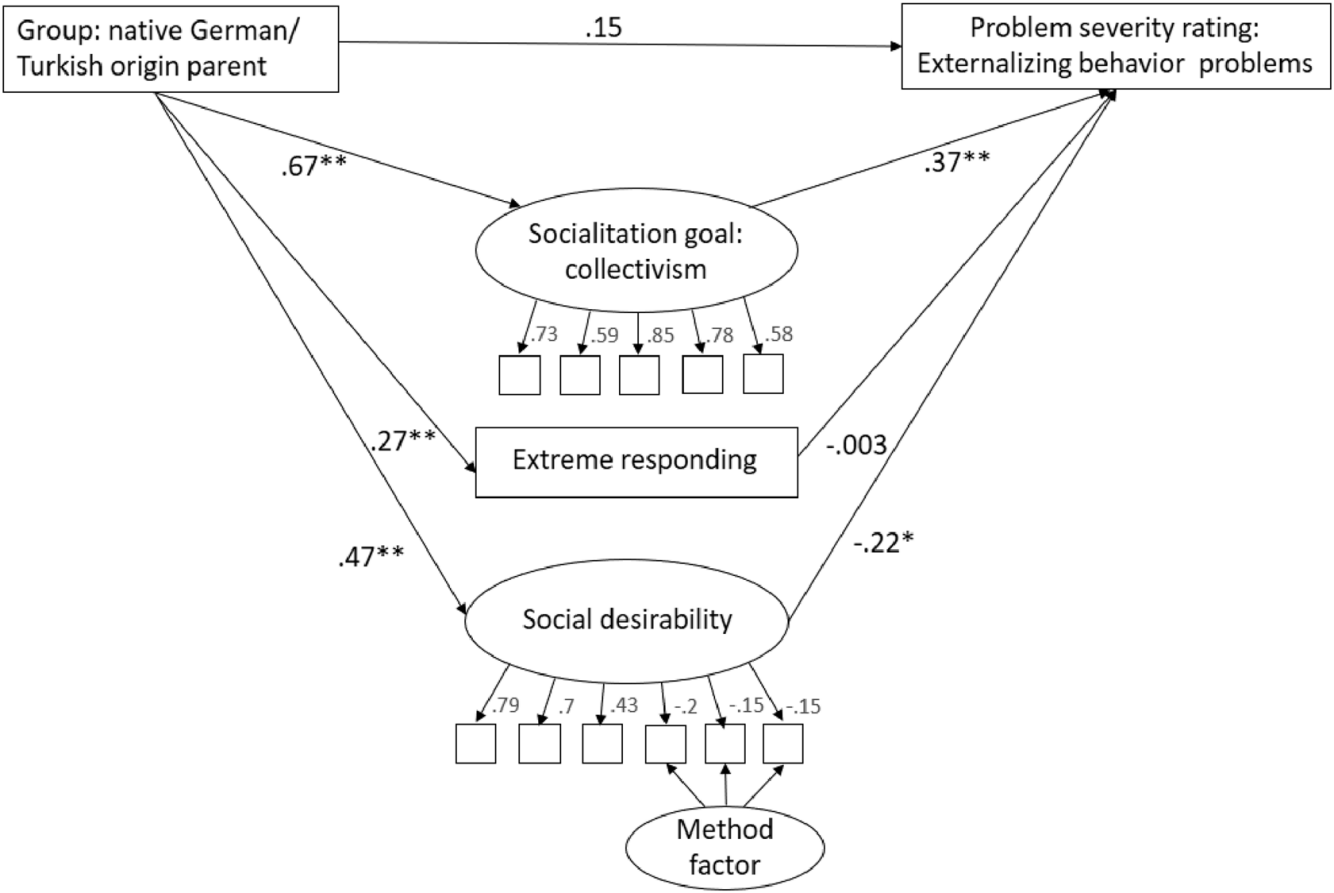
Mediation model with collectivism as mediator. Standardized path coefficients (*β*) are dispayed. **p  < 0.01; *p  < 0.05

The relation between group (native German parents/Turkish origin parents) and problem severity rating of the externalizing problem behavior vignettes was fully mediated by the agreement with the socialization goals obedience (*χ*^2^(57) = 83.0, *p* = 0.01; mediation path a*b: *β* = 0.22, *p* < 0.001) and collectivism (χ^2^(65) = 98.58, *p* = 0.01; mediation path a*b: *β* = 0.25, *p* < 0.001. Path coefficients are shown in Fig. 1 for obedience and Fig. 2 for collectivism as a mediator. Turkish origin parents showed a higher level of social desirability (*β* = 0.47, *p* < 0.001) and a more extreme response style (*β* = 0.27, *p* < 0.001). Neither social desirability (*β* = − 0.2, *p* = 0.06) nor extreme response style (*β* = − 0.03, *p* = 0.68) predicted problem severity ratings in the obedience mediation model. Social desirability did predict problem severity rating in the collectivism model (*β* = − 0.22, *p* = 0.044), but there was no mediation effect (mediation path a*b: *β* = − 0.11, *p* = 0.07). Extreme response style did not predict severity ratings in the collectivism model (*β* = − 0.003, *p* = 0.98).

Since both collectivism and obedience fully mediated the effect in the two separate models, it seems likely that the joint variance of the two variables mediates the effect. To further investigate this assumption, we built three new variables: (1) one representing the shared variance of collectivism and obedience (predicted values when collectivism is regressed on obedience), (2) one representing the unique variance of collectivism (residual of the regression of collectivism on obedience), and (3) representing the unique variance of obedience (residual of the regression of obedience on collectivism). We tested two regression models with the shared variance as one predictor and the unique variance of one of the socialization goals as the second predictor and the problem severity rating as the outcome. In both models, the shared variance variable had a significant effect on problem severity ratings (*β* = 0.41/*β* = 0.45; *p* < 0.001), while neither the unique variance of collectivism nor the unique variance of obedience explained any additional variance in the problem severity ratings (unique variance of collectivism: *ß* = 0.05, *p* = 0.47; unique variance of obedience *β* = -0.07, *p* = 0.47). See supplemental materials online for detailed results (Table A6).

### Additional Analyses

#### Self-Development as a Mediator

Turkish origin and native German parents did not differ in their agreement with the socialization goal self-development (*β* = − 0.009, *p* = 0.94) and agreement with the goal of self-development did not predict the problem severity rating of vignettes describing internalizing problem behavior (*β* = 0.13, *p* = 0.29) or externalizing problem behavior (*β* = 0.03, *p* = 0.68).

#### Role of Immigrant Generation and Length of Stay in Germany

There were no correlations between problem severity ratings of internalizing problem behavior vignettes and immigrant generation (*r* = 0.038, *p* = 0.75) or length of stay in Germany (*r* = − 0.008, *p* = 0.96) and none between severity ratings of externalizing problem behavior vignettes and immigrant generation (*r* = − 0.03, *p* = 0.83) or length of stay in Germany (*r* = − 0.15, *p* = 0.32).

## Discussion

Researchers, prevention planners and practitioners in culturally diverse societies usually assume that they can use screening instruments for children’s mental health for all parents without having to adjust scale values for specific cultural characteristics. For this assumption to be valid, parents must have comparable ideas about when a certain behavior is problematic.

We compared how problematic native German and Turkish origin parents in Germany rated internalizing and externalizing problem behavior described in vignettes. Whereas there was no difference in the rating of internalizing problem behavior, Turkish origin parents rated the behavior described in the externalizing problem behavior vignettes as more problematic than native German parents. The lack of comparability between parental ratings in different cultural groups has been shown in previous studies [9–11]. As we parallelized our samples by education, gender and age of the child and the respondent, partnership status and urban/rural residential area*,* these characteristics cannot explain the difference in ratings. Turkish origin parents rating the same externalizing behavior as more problematic than native German parents has important implications for the interpretation of parental reports. When planning intervention and prevention for groups with specific needs, researchers have to consider a potential over- (or under-) estimation of problem behavior in specific groups. It is possible that differences in problem behavior between immigrant and non-immigrant groups in Germany found in previous studies (e.g., [4, 6]) are biased. Individual diagnostic decisions based on screening questionnaires may also be influenced. However, taking a closer look at the mild, medium and severe externalizing problem behavior ratings in our study, we found that a significant effect is carried by the rating of the mild vignette. Parents did not differ in the rating of the severe problem behavior vignette. Presumably there will be no bias in clinically relevant symptoms. Runge and Soellner [10] also found no difference in the predictive power of the screening instrument SDQ for clinical diagnoses between native German and Turkish origin parents in Germany, although the total score of the instrument was not comparable between the two groups.

We expected the difference between groups only to emerge in older children. This hypothesis was based on findings that Turkish origin parents expect their children to reach specific developmental steps later than native German parents [41]. Correspondingly, Turkish parents show a more indulgent and permissive parenting style during their children’s early childhood, when the child is not yet believed to be responsible for its actions, compared to later childhood [24, 27, 42]. We did not find an effect of children’s age on problem severity ratings. It is possible that the effect was not found because, with our age range between 4 and 12 years of age, the phase of indulgence in Turkish parents is almost up.

### The Mediating Effect of Socialization Goals

The group difference in externalizing problem behavior ratings was mediated by the approval of the socialization goals obedience and collectivism. Turkish origin parents valued obedience and collectivism more highly than native German parents. This finding fits with previous studies [18, 25, 27] and was expected, since Turkish origin parents were assumed to be allocated to the interdependent cultural model or to the model of autonomous relatedness [21]. Parents who value obedience and collectivism more, rated the externalizing problem behavior in the vignettes describing behavior opposing these values as more problematic. In line with Durgel [26], we did not find effects of immigrant generation or length of stay in Germany on problem severity ratings. She explains the finding by Turkish immigrants having close contact with the Turkish community in Germany, living in neighborhoods with many other Turkish immigrants or engaging in Turkish organizations in their free time in Germany [26]. Thus length of stay does not mirror acculturation or adaption to the host country, since immigrants can also choose a separated or marginalized acculturation strategy [43].

We additionally included social desirability and extreme responding as response styles in our analysis. While Turkish origin parents scored higher on both, these variables did not mediate the relation between group and severity rating nor did they change the mediating effect of obedience and collectivism (see online supplementary material Figs. A1 and A2 for results without the variables social desirability and extreme responding). However, since differences between the groups are observable, social desirability and extreme responding should be taken into account when evaluating parental reports in future studies.

We tested the mediating effect of collectivism and obedience in two separate models and both socialization goals fully mediated the effect of cultural group on severity ratings. We therefore assume that it is the shared variance of both constructs that mediates the effect. In an exploratory additional analysis, we found some evidence for this assumption. It should furthermore be investigated if the socialization goals obedience and collectivism are independent constructs in different cultural groups. In our sample, collectivism and obedience correlated very highly, which calls into question whether they should be assumed to be independent constructs. Moreover, we translated the scales for this study and only tested their factorial structures in a relatively small sample. Further studies with larger, representative samples should investigate the factor structures.

We did not find a difference in the valuation of self-development between native German and Turkish origin parents. This suggests that we can assign our Turkish origin sample to the cultural model of autonomous-relatedness, as in this model self-development is as important as in the independent model, while parents still value close relations (collectivism). In contrast to our study, in the studies by Citlak et al. [24] and Durgel [26], native German mothers placed more emphasis on self-control and autonomy as socialization goals than Turkish origin mothers in Germany. Since our sample is highly educated with a majority of 1st generation migrants being in Germany for an average of less than 10 years, it might comprise the Turkish urban, high educated people Kagitcibasi and Ataca [21] described as autonomous-related. The samples in the studies by Citlak et al. [24] and Durgel [26] however, might include more Turkish origin mothers being born in Germany (2nd generation) who maintain the values of the independent cultural model of their parents and grandparents.

## Limitations

We used vignettes to examine differences in the problem perception of parents. This presupposes that vignettes are interpreted by readers in the same way (vignette equivalence) and that respondents respond to the vignettes in the same way as they would, in our case, report about their children (response consistency; [44]). We used vignette ranking as a test for vignette equivalence. As we found the ranking (mild, medium and severe problem behavior) in both groups as expected, we are confident that the vignettes were interpreted as intended. We did not test response consistency. Still, we assume that response consistency is easier to achieve in our case, as vignettes were not used for self-reports, but for reports about the respondent’s child. Thus, respondents have to think of their child and remember their child’s behavior both when responding to a screening instrument and when responding to the vignette. The response process should be more similar. Nevertheless, our findings should be replicated using other methods, ideally observation studies to objectively evaluate a child’s behavior and compare the objective rating with parental reports.

Our Turkish origin parents sample had a higher level of education and consisted of more 1st generation immigrants than the population of Turkish origin people in Germany. It is thus not representative for the group. However, we did not find an effect of immigrant generation on problem severity ratings in our data. Regarding education, we would expect lower educated respondents to value obedience more than higher educated respondents, thus we would expect an even bigger effect in a more representative sample. We tested our hypothesis in only one country (Germany) and by only including two cultural groups (native German and Turkish origin parents). To ensure the generalizability of findings, a cross-country study including diverse cultural groups is needed.

Finally, as in most cross-sectional research, the causal effect of the socialization goals on problem severity ranking can only be assumed but cannot be proved. Longitudinal or experimental studies would provide stronger evidence of causality. However, we believe that the direction of causality we propose is the theoretically more sensible one.

## Summary

Children of immigrants are at increased risk of developing mental health problems. To assess the mental health of younger children, parental reports are usually applied. However, it has been called into question whether widely used screening instruments for child mental health can provide comparable results across countries and cultures. So far, little research has been done on the factors that lead to this lack of comparability. One mechanism could be the different socialization goals of parents from different cultures. Socialization goals can influence whether and to what extent a parent considers a behavior to be problematic. We tested comparability of parental reports using a vignette approach. Parents were asked to rate the perceived problem severity of the same behavior depicted in vignettes. We expected and found that parents of Turkish origin in Germany rate the externalizing problem behavior depicted in the vignettes as more problematic compared to native German parents. The effect was fully mediated by approval of the socialization goals obedience and collectivism. We found the mediation when controlling and when not controlling for social desirability responding and an extreme response style. There was no difference in the rating of internalizing problem behavior depicted in the vignettes. Our study underlines the problems of validity when comparing parenting reports between different cultural groups. We replicated the findings from previous studies [9, 11] using another method, the vignette approach. To the best of our knowledge, this is the first study to investigate the underlying reasons for the lack of comparability. We found that the consideration of parental socialization goals could help researchers and practitioners when classifying parental reports in cross-cultural settings.

### Supplementary Information

Below is the link to the electronic supplementary material.

Supplementary file1 (DOCX 51 kb)
