# Supplementary material for: Cultural Bias in Parent Reports: The Role of Socialization Goals When Parents Report on Their Child’s Problem Behavior
Source: Child Psychiatry Hum Dev. 2022 Nov 12;55(4):1020–30. doi: 10.1007/s10578-022-01464-y (PMC11245439; doi:10.1007/s10578-022-01464-y)

**Online supplementary material**

| **Table A1**  *Bivariate correlations of variables* | | | | | | |
| --- | --- | --- | --- | --- | --- | --- |
|  | 1 | 2 | 3 | 4 | 5 | 6 |
| 1. collectivism |  |  |  |  |  |  |
| 2. obedience | 0.882** |  |  |  |  |  |
| 3. self-development | 0.668* | 0.589* |  |  |  |  |
| 4. Social Desirability | 0.388** | 0.253* | 0.464 |  |  |  |
| 5. Extreme responding | 0.081** | 0.081** | 0.107 | 0.053** |  |  |
| 6. Problem Severity rating: externalizing problem behaviour | 0.575** | 0.747** | 0.342* | -0.085 | 0.034 |  |
| 7. Problem Severity rating: internalizing problem behaviour | -0.036 | -0.032 | 0.194 | -0.097 | 0.024 | 0.657** |
| **p*<.05, ***p*<.01 |  |  |  |  |  |  |

**Table A2**

*Measurement invariance testing for the socialization goal collectivism*

|  | Configural | Weak | Strong | Strong  Partial^1^ |
| --- | --- | --- | --- | --- |
| Degrees of freedom | 8 | 12 | 16 | 13 |
| $\chi^{2}$ | 5.98 | 10.29 | 53.0** | 14.56 |
| $\Delta\chi^{2}$ |  |  | 42.71** | 3.82 |
| $\chi^{2}$ GN sample | 2.58 | 4.44 | 25.03 | 5.90 |
| $\chi^{2}$ TO sample | 3.41 | 5.85 | 27.96 | 8.65 |
| CFI | 1.0 | 1.0 | .77 | 0.99 |
| $\Delta$CFI |  | .00 |  | 0.008 |
| RMSEA | .00 | .00 | .16 | .04 |
| $\Delta$RMSEA |  | .00 | .16 | .04 |
| SRMR | .03 | .05 | .14 | .063 |

*Note.* GN = German native, TO = Turkish Origin. $\Delta\chi^{2}$ and $\Delta$CFI refers to change from configural to weak and from weak to strong invariance.

**p* < 0.05; ***p* < 0.01; $\Delta\chi^{2}$ tested by using ANOVA

^1^Partial invariance collectivism scale: three intercepts were freed

| **Table A3**  *Measurement invariance testing for the socialization goal obedience* | | | |
| --- | --- | --- | --- |
|  | Configural | Weak | Strong |
| Degrees of freedom | 4 | 7 | 10 |
| $\chi^{2}$ | 1.01 | 4.28 | 11.29 |
| $\Delta\chi^{2}$ |  | 3.27 | 7.01 |
| $\chi^{2}$ GN sample | 0.43 | 1.41 | 3.2 |
| $\chi^{2}$ TO sample | 0.58 | 2.87 | 8.09 |
| CFI | 1.0 | 1.0 | 0.99 |
| $\Delta$CFI |  | 0.0 | 0.01 |
| RMSEA | 0.00 | 0.0 | 0.04 |
| $\Delta$RMSEA |  | 0.0 | 0.04 |
| SRMR | 0.02 | 0.05 | 0.07 |

*Note.* GN = German native, TO = Turkish Origin. $\Delta\chi^{2}$ and $\Delta$CFI refers to change from configural to weak and from weak to strong invariance.

**p* < 0.05; ***p* < 0.01; $\Delta\chi^{2}$ tested by using ANOVA

**p* < 0.05; ***p* < 0.01

| **Table A4**  *Measurement invariance testing for the socialization goal self-development* | | | | |
| --- | --- | --- | --- | --- |
|  | Configural | Weak | Weak Partial^1^ | Strong Partial^2^ |
| Degrees of freedom | 4 | 7 | 6 | 7 |
| $\chi^{2}$ | 3.32 | 14.18* | 3.24 | 3.87 |
| $\Delta\chi^{2}$ |  | 10.86** | 0.08 | 0.63 |
| $\chi^{2}$ GN sample | 2.2 | 2.34 | 2.02 | 2.30 |
| $\chi^{2}$ TO sample | 1.12 | 11.84 | 1.22 | 1.56 |
| CFI | 1.0 | 0.87 | 1.0 | 1.0 |
| $\Delta$CFI |  | 0.13 | 0.0 | 0.0 |
| RMSEA | 0.0 | 0.104 | 0.0 | 0.0 |
| $\Delta$RMSEA |  | 0.104 | 0.0 | 0.0 |
| SRMR | 0.03 | 0.062 | 0.03 | 0.03 |

*Note.* GN = German native, TO = Turkish Origin. $\Delta\chi^{2}$ and $\Delta$CFI refers to change from configural to weak and from weak to strong invariance.

**p* < 0.05; ***p* < 0.01; $\Delta\chi^{2}$ tested by using ANOVA

^1^Partial invariance: one loading was freed; ^2^one loading and two intercepts were freed

| **Table A5**  *Measurement invariance testing for the social desirability scale (KSE-G)* | | | | |
| --- | --- | --- | --- | --- |
|  | Configural | Weak | Strong | Strong partial^1^ |
| Degrees of freedom | 12 | 19 | 23 | 22 |
| $\chi^{2}$ | 10.29 | 17.36 | 25.64 | 20.18 |
| $\Delta\chi^{2}$ |  |  | 8.28* | 2.82 |
| $\chi^{2}$ GN sample | 6.97 | 7.02 | 11.38 | 9.32 |
| $\chi^{2}$ TO sample | 3.32 | 10.34 | 14.27 | 10.87 |
| CFI | 1.0 | 1.0 | 0.97 | 1.0 |
| $\Delta$CFI |  | 0.0 | 0.03 | 0.0 |
| RMSEA | 0.0 | 0.0 | 0.04 | 0.0 |
| $\Delta$RMSEA |  | 0.0 | 0.04 | 0.0 |
| SRMR | 0.04 | 0.06 | 0.07 | 0.07 |

*Note.* GN = German native, TO = Turkish Origin. $\Delta\chi^{2}$ and $\Delta$CFI refers to change from configural to weak and from weak to strong invariance.

**p* < 0.05; ***p* < 0.01; $\Delta\chi^{2}$ tested by using ANOVA

^1^Partial invariance: one intercept was freed

**Table A6**

*Regression models containing the shared variance of obedience and collectivism and the unique variance of each collectivism and obedience as predictors of externalizing vignette problem severity ratings*

|  | **F** | **R²** | ***β*** |
| --- | --- | --- | --- |
| **Model 1** | 17.41** | 0.17 |  |
| Shared Variance |  |  | .41** |
| Unique Variance collectivism |  |  | .05 |
| **Model 2** | 17.41** | 0.17 |  |
| Shared Variance |  |  | .45** |
| Unique Variance obedience |  |  | -.07 |
| *p<.05, **p<.01 | | | |

**H2: Mediation models without taking into account social desirability and extreme responding**

The relation between group (native German parents/Turkish origin parents) and problem severity rating of the externalizing problem behaviour vignettes was fully mediated by the agreement to the socialization goals obedience (*χ*²(8)=9.66, *p*=.29; mediation path a*b: *β*=.22, p=.001; total effect *β* =.29, p<.001) and collectivism (χ²(9)=7.62, *p*=.57; mediation path a*b: *β* =.21, p<.001; total effect *β*=.29, p<.001). Path coefficients are shown in Figure A1 for obedience and Figure A2 for collectivism as mediator.

**Figure A1**

*Mediation model with obedience as mediator*


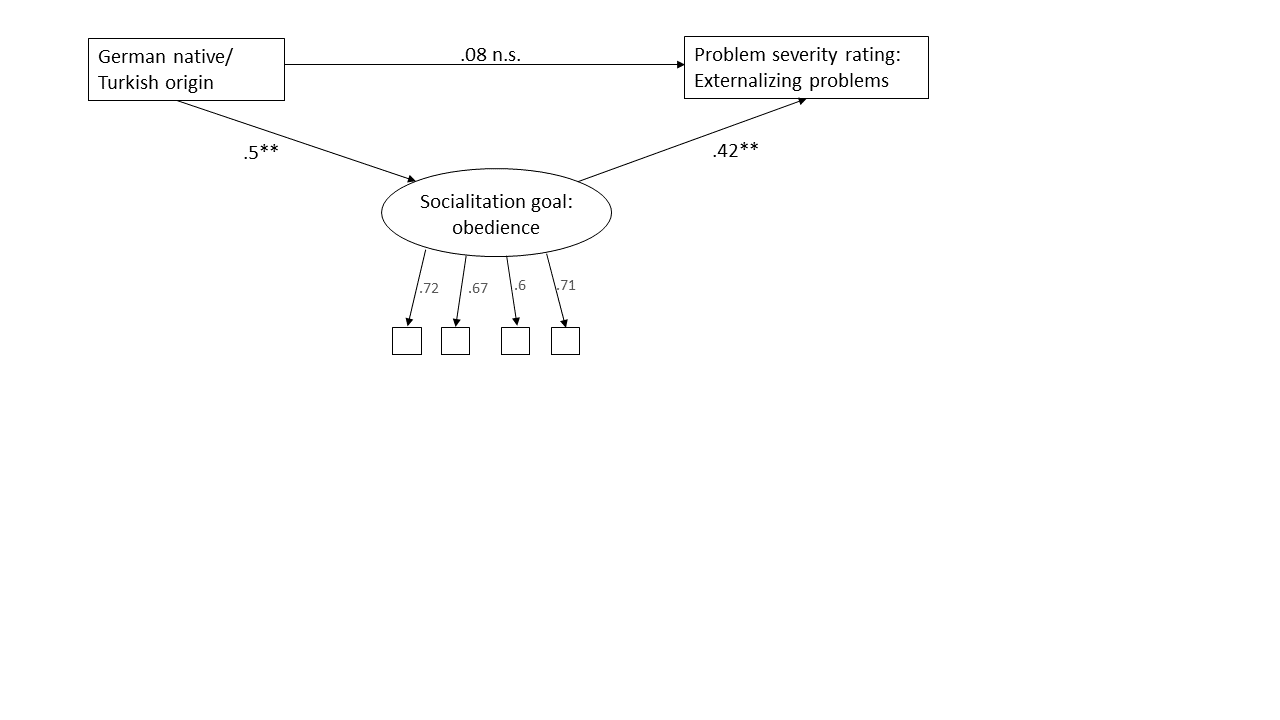


**Figure A2**

*Mediation model with collectivism as mediator*


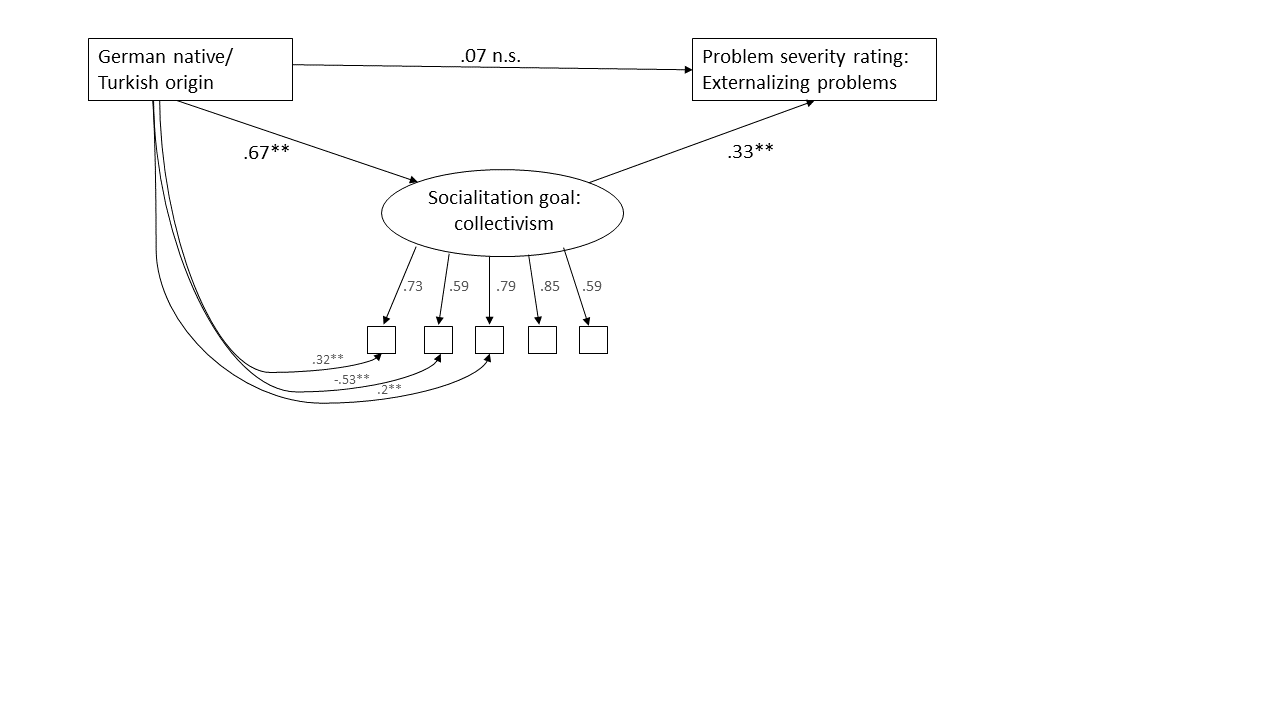

Supplement: Supplementary file 1 — Supplementary file1 (DOCX 51 kb) [file 10578_2022_1464_MOESM1_ESM.docx]
